# Evaluation of droplet digital qRT-PCR (dd qRT-PCR) for quantification of SARS CoV-2 RNA in stool and urine specimens of COVID-19 patients

**DOI:** 10.3389/fmed.2023.1148688

**Published:** 2023-06-26

**Authors:** Manohar Shinde, Mallika Lavania, Jatin Rawal, Nutan Chavan, Pooja Shinde

**Affiliations:** Enteric Viruses Group, ICMR-National Institute of Virology, Pune, Maharashtra, India

**Keywords:** droplet digital PCR, N-gene, real time qRT-PCR, SARS-CoV-2, stool, urine

## Abstract

**Introduction:**

There have been a few reports of viral load detection in stool and urine samples of patients with coronavirus disease 2019 (COVID-19), and the transmission of the virus through faecal oral route. For clinical diagnosis and treatment, the widely used reverse transcription-polymerase chain reaction (qRT-PCR) method has some limitations.

**Methods:**

The aim of our study to assess the presence and concentration of SARS CoV-2 RNA in stool and urine samples from COVID-19 patients with mild, moderate, and severe disease, we compared a traditional qRT-PCR approach with a ddPCR. ddPCR and qRT-PCR-based target gene analysis were performed on 107 COVID-19-confirmed patients paired samples (N1 and N2). The MagMax magnetic beads base method was used to isolate RNA. Real-time qRT-PCR and dd PCR were performed on all patients.

**Results and Discussion:**

The average cycle threshold (Ct) of qRT-PCR was highly correlated with the average copy number of 327.10 copies/l analyzed in ddPCR. In ddPCR, urine samples showed 27.1% positivity while for stool it was 100%.

**Conclusion:**

This study’s findings not only show that SARS CoV-2 is present in urine and faeces, but also suggest that low concentrations of the viral target ddPCR make it easier to identify positive samples and help resolve for cases of inconclusive diagnosis.

## 1. Introduction

On December 31, 2019, China reported the first cases of pneumonia from an unidentified source to the World Health Organization (WHO), and on March 11, 2020, WHO declared the coronavirus disease of 2019 (COVID-19) as a pandemic. Over 6.4 million fatalities and 605 million confirmed cases of COVID-19 infections had been documented globally as of September 11^th^, 2022 (1). It has been almost 3 years since the COVID-19 pandemic started and it continues to affect the global population, as new strains of the virus keep emerging. In spite of developing newer treatment and diagnostic modalities, including very effective vaccines, the disease remains one of the major challenges countries across the globe face today. The World health organization estimates that till now about 400 million people have had the disease and 5 million have died because of its complications. This large number indicates the rapid transmission of this disease, which has been proven to spread through more than one route. It is known that the SARS CoV-2 presents differently in infected people, it can range from asymptomatic or mild respiratory infection to severe pneumonia with acute respiratory distress syndrome or multi organ failure, which might have a fatal outcome. Among those who develop symptoms the majority present with symptoms of fever, cough, fatigue, myalgia and rhinitis. A significant proportion of infected people also present with gastrointestinal symptoms including diarrhoea, abdominal pain, and vomiting. Occasional observation of dominance of gastro-intestinal symptoms without any respiratory symptoms have also been noted, the possible reason proposed for this finding is the circulation of two types of SARS CoV-2, one with gut tropism and another with lung tropism.

The gold standard method to assess genomic or complimentary DNA levels is quantitative PCR (qPCR), but without proper sample and primer validation and verification, the resulting data might be very varied, false, and impossible to reproduce. Poor data quality has its origins in the insufficient dilution of chemical and protein impurities that, in varying degrees, block Taq polymerase and primer annealing. The samples with the lowest expression differences of twofold or less and the least numerous targets are the most vulnerable, frustrating, and frequently most intriguing. In this study, Droplet Digital PCR (ddPCR) and quantitative PCR (qPCR) systems were directly compared for the detection of gene expression in well-characterized samples utilizing small amounts of pure, synthetic DNA under the same reaction conditions. Quantitative Real-time reverse transcriptase polymerase chain reaction (qRT-PCR) detection of SARS CoV-2 RNA in nasopharyngeal swabs is used to diagnose the majority of COVID-19 cases (The qRT-PCR technology has two advantages: high throughput and sensitivity). Numerous testing platforms have received FDA and CE IVD approval and have been clinically used to diagnose SARS CoV-2 infection as of the first quarter of 2021. These point-of-care tests are quick, but many of them have low sensitivity and high false-negative rates, as a disadvantage or better which limit their use (2). qRT-PCR technology may detect small amounts of virus with high throughput, although faint positives Ct > 35 may be challenging to separate from technical artifacts. The current gold standard for the etiological diagnosis of COVID-19 is viral nucleic acid detection by reverse transcription PCR (RT-PCR) which targets viral genes such ORF1a/b, E, S, and N genes. The sensitivity and accuracy of RT-PCR, however, have been questioned because some patients who had a high degree of clinical suspicion for the disease based on their exposure history and clinical presentation had negative results as well as positive findings in some confirmed cases after recovery (3, 4). Additionally, the RT-PCR technique is unable to assess the efficacy of antiviral medications and has limitations on viral load analysis for determining disease progression and prognosis. Droplet digital PCR (ddPCR) has the benefit of absolute quantification and is more sensitive for virus identification than RT-PCR, according to a number of studies (5, 6).

ddPCR is an orthogonal technique that can be used to detect and measure accurate nucleic acid copy numbers as well as incredibly low amounts of nucleic acid. Several investigations have shown that ddPCR could detect SARS CoV-2 RNA in various body fluids, such as plasma (7, 8).

ddPCR is a very sensitive PCR technique for absolute nucleic acid quantification without the need for a reference curve. Although ddPCR utilization in research labs has grown over the past 10 years, this method is rarely employed in clinical labs, mostly because of its high cost (9).

In order to assess the presence and concentration of SARS CoV-2 RNA in stool and urine samples from COVID-19 patients with mild, moderate, and severe disease, we compared a traditional qRT-PCR approach typically used in clinical microbiology laboratories with a ddPCR.

## 2. Materials and methods

### 2.1. Patients and sampling

**Figure 1:**
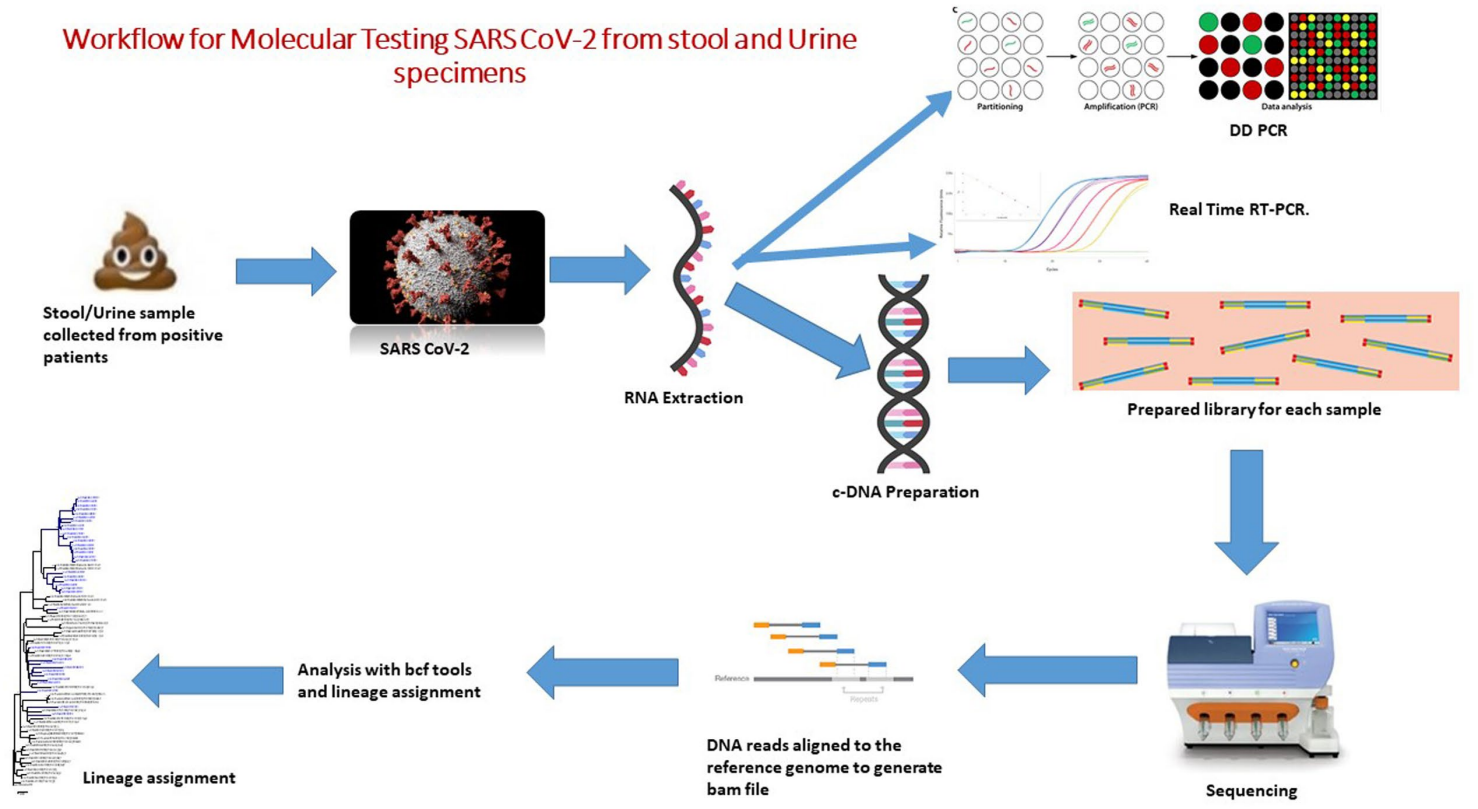
Workflow for molecular testing SARS CoV-2 from stool and urine specimens.

All of the registered patients were recruited from the different hospitals in the Pune, Deenanath Mangeshkar, Jehangir, and Lokmaanya hospitals in Pune, Western India, between May 2020 and August 2021. Real-time reverse transcription polymerase chain reaction (**qRT-PCR**) results on oro/nasopharyngeal swab samples showed that all of the registered patients were positive for SARS CoV-2 RNA. The study was approved by the Institutional Ethics Committee of ICMR-National Institute of Virology, Pune, Maharashtra, India (No. NIV/IEC/June/2020/D-14 dated 24^th^ June 2020). Workflow for the molecular diagnosis of SARS CoV-2 from stool and urine specimens was represented in Figure 1.

In total, 107 patients were enrolled in the study. Patients who had been diagnosed with COVID-19 in a lab had their faeces and urine samples taken. Prior to being tested for SARS CoV-2, all hospital samples were transported to the ICMR-National Institute of Virology in Pune and stored at −20°C. Samples were stored at −20°C for up to 3 days and subsequently transferred to −80°C until analysis. We included twenty normal samples as a control to check the specificity of assay.

### 2.2. Sample processing

To remove debris, 30% faecal suspensions in 0.01 M phosphate buffered saline (PBS), pH 7.4, were centrifuged at 4000 rpm (Hettich Universal 320R centrifuge) for 10 min. 10 mL of urine sample was collected in a 15 mL sterile tube. Centrifugation was performed at 500 g for 20 min at 4°C. The supernatant was removed, and the pellet was used to extract RNA (Figure 1).

### 2.3. RNA extraction

The viral RNA was extracted from 30% (w/v) suspensions of faecal and urine samples using spin columns and the Qiagen Viral RNA extraction Kit (Cat No52904) as directed by the manufacturer (Qiagen, Hilden, Germany).

### 2.4. Construction of RNA standards

Forward primers with a T7 promoter tag at the 5′ end were created to amplify full-length E gene and N gene sections because the whole SARS CoV-2 genome was taken from the public database and primers were designed. To obtain the desired PCR result, gene-specific PCR was conducted. Amplicons were cleaned by using Qiagen direct PCR purification kit (Cat No- 28104 Qiagen, Hilden, Germany). *In Vitro* Transcribed (IVT) RNA was synthesized using T7 Riboprobe® Systems (Cat No: P1440, Promega, United States) in accordance with the kit’s instructions. Each IVT RNA product was serially diluted 10 times before being tested for specific detection and determination of limit of detection using the appropriate gene primer probe sets (10).

The concentration of synthetic fragment of transcribed RNA was measured by fluorometric analysis (Qubit, Thermo Scientific), and then standard curve was constructed by using tenfold serial dilutions of RNA. The copy numbers of the standard RNAs ranged from 2.5 to 2.5 x 10^8^/xuL, were used for the consctruction of standard curve for absolute quantification in qRT-PCR. After standardizing the qRT-PCR data using the standard curve in the instrument software (CFX96^™^ thermocycler), the Ct value for both genes was determined (Bio-Rad, Hercules, California, United States). For data comparison, the Ct of each analysis was taken into consideration.

### 2.5. Real-time qRT-PCR assay based on N1 and N2 gene

Using a CFX96^™^ thermocycler and the Qiagen SARS CoV-2 N1 + N2 assay kit (Cat. No. 222015, Qiagen, Germany), qRT-PCR was carried out with 5 μL of total RNA isolated from stool and urine samples (Qiacuity QX-200, Qiagen, Germany). The N1 and N2 genes, which code for the viral nucleocapsid, the E gene, which codes for the viral envelop, as well as the RNAse P gene as an internal control, are all detected by this kit. In accordance with the manufacturer’s recommendations, samples were deemed positive for SARS CoV-2 if any of the genes (E or N) it detects had a Ct value below 37.

All the twenty normal samples showed negative results by using Q-PCR targeting this multiplex N1 + N2 assay kit.

### 2.6. Droplet digital qRT-PCR (dd qRT-PCR) assay based on N1 and N2 gene

SARS CoV-2 RNA was detected and quantified in 5 μL of total RNA obtained from stool and urine specimens using the SARS CoV-2 N1 + N2 assay kit according to manufacturer’s instructions on a QX-200 ddPCR platform (Qiacuity QX-200, Qiagen, Germany) and a recent published literature on waste water (11). The SARS CoV-2 CoV-2 N1 + N2 Assay is a mixture of four primers and two probes purified by HPLC at a 20x concentration. These four primers are based on the CDC design, targeting the regions N1 and N2 of the viral genome. The two probes are coupled with FAM as a reporter dye and use ZEN^™^ quenchers for enhanced sensitivity. For the N1 and N2 assays, the concentrations of the primer and probe, as well as the annealing temperature and duration, were optimized. N1 and N2 assays were carried out in 40 μL reaction mixtures using the QIAcuity One-Step Viral qRT-PCR Kit (Cat no. 1123145, Qiagen) on 26,000 24-well Nanoplates under ideal circumstances (catalog no. 250001, Qiagen). The microfluidic dPCR plates 26,000 QIAcuity 24-well Nanoplates enable 24 samples to be run with up to 26,000 partitions/well. Each partition has a volume of 0.91 nL and the PCR takes place within each partition.

### 2.7. Statistical analysis

The Mann–Whitney U test was used to make comparisons between the two groups. The Spearman correlation test was used to examine the relationship between the Ct values of qRT-PCR and the viral load determined by ddPCR. Statistical significance was defined as a *p* value less than 0.05 (two sided). The analyzes described above were carried out with either Prism 7.0 (GraphPad, La Jolla, CA, United States) software.

## 3. Results

### 3.1. Baseline demographic characteristics of patients

**Figure 2:**
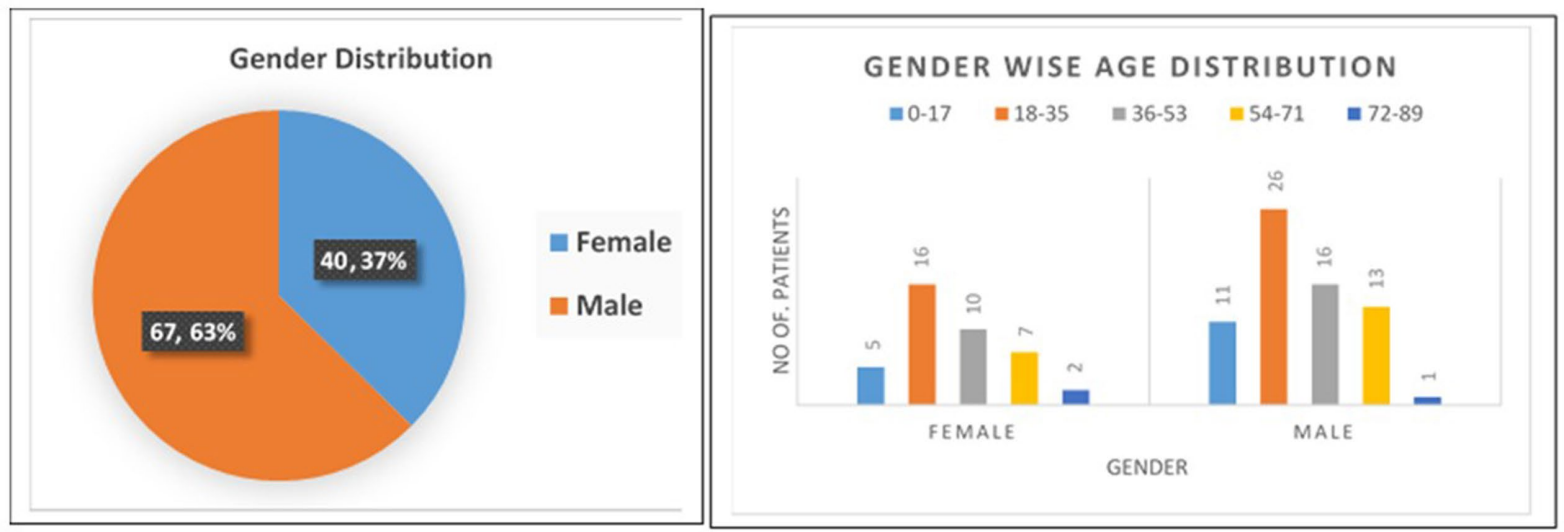
Baseline demographic and clinical characteristics of the study population.

In this study, 107 COVID-19 positive patients confirmed by real time qRT-PCR from all age groups who were admitted in different COVID Care Center of Pune District were enrolled, in which 40 (37%) were female, & 67 (63%) were male. The demographic and clinical details of the patients are described in Figure 2. According to the age distribution, the median age was 32 years, with 16 participants belonging to the 0–17 age range, 42 participants to the 18–35 age range, 26 participants to the 36–53 age range, 20 participants to the 54–71 age range, and 3 participants to the 72–89 age range. Most of the participants (68 patients) who had COVID-19 infection were in the 18–35, 36–53, and 0–17 age range.

At the time of admission, fever (78.50%), cough (58.88%), loss of taste or smell (43.93%), diarrhoea (33.64%), sore throat (27.10%), nausea and vomiting (26.17%), runny nose (24.30%), bloody sputum (16.82%), chest discomfort (14.95%), and abdominal pain (14.02%) were the signs and symptoms that were most prominent. Among these 107 participants, 19 (18%) participants were in close contact with known positive case of COVID-19 patient, while 88 (82%) were not having any close contact with known case in last 14 days.

After admission to the COVID Care Center, stool & urine specimens were collected from the patient from day 0, i.e., day on which patient was admitted, while maximum number of specimens were collected on Day 1, 2, 3, and 6.

### 3.2. Performance of the assays

**Figure 3:**
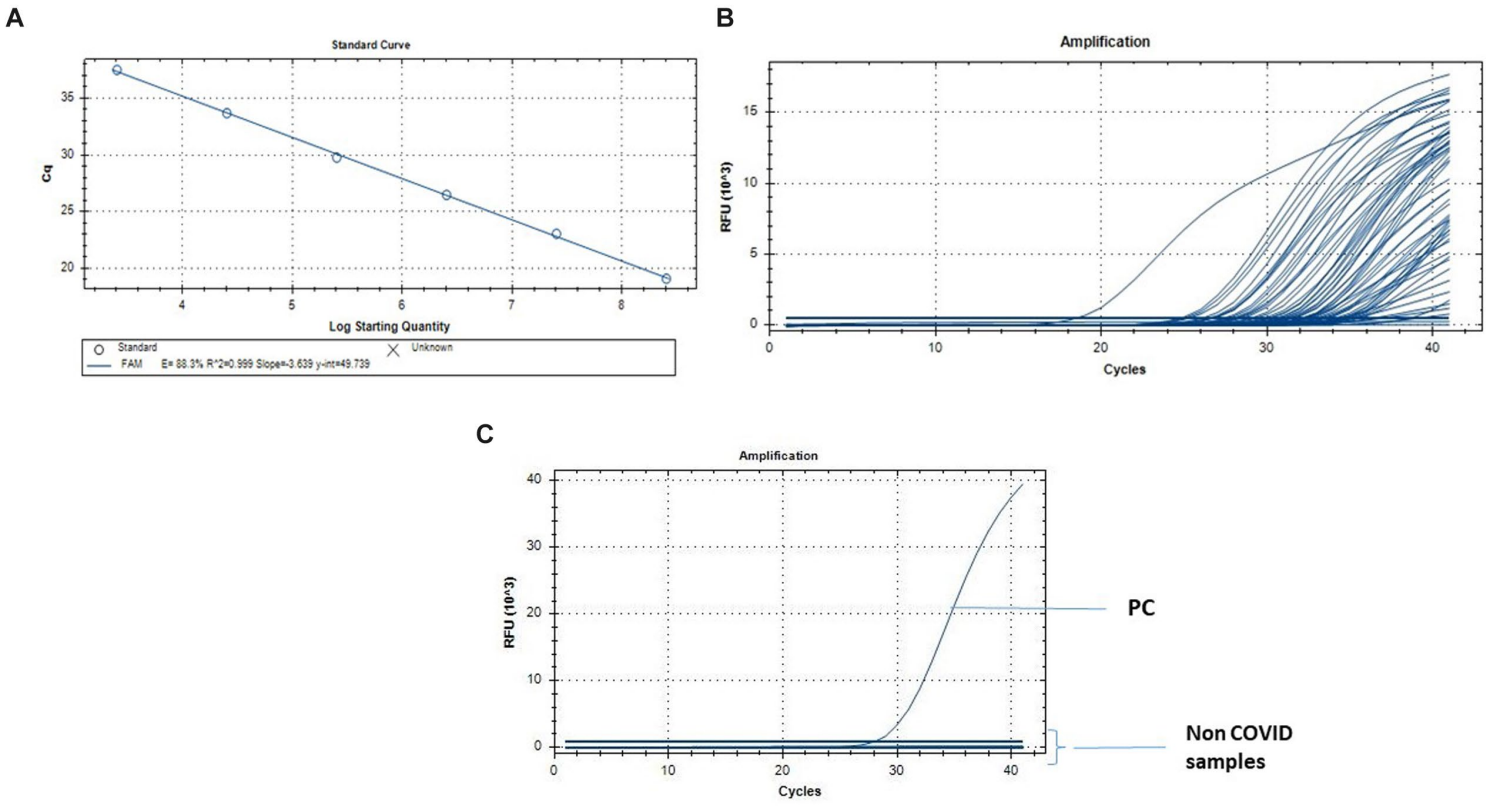
Quantification of SARS-CoV-2 by qRT-PCR. **(A)** Standard curve for the real-time PCR targeting N gene. The X-axis represents copies of the plasmids, and the Y-axis represents the cycle threshold (Cq). The assays were linear from 2.5 × 10^8^ to 2.5 gene copies/reaction. **(B)** q-RT PCR results from the fecal and urine specimens of the individuals infected with SARS CoV-2\u00B0C. q-RT PCR from the Non COVID-19 normal individuals for the specificity of the assay.

The quantification for the N1 and N2 qRT-PCR standard curves ranged from 2.5×10^8^ to 2.5 gene copies/reaction. The approach revealed a strong linear correlation (R2 = 0.999) between predicted and actual SARS CoV-2 measurement (Figure 3).

The amplification efficiencies, y-intercepts and the correlation coefficient (r^2^) were 88.3%, 49.73 and 0.999 for N gene assay (Figure 3). The qRT-PCR assay limits of detection was 1.8-gene copies/reaction for N1 + N2 assay.

### 3.3. Comparative analysis of qRT-PCR and ddPCR in stool and urine specimens

**Figure 4:**
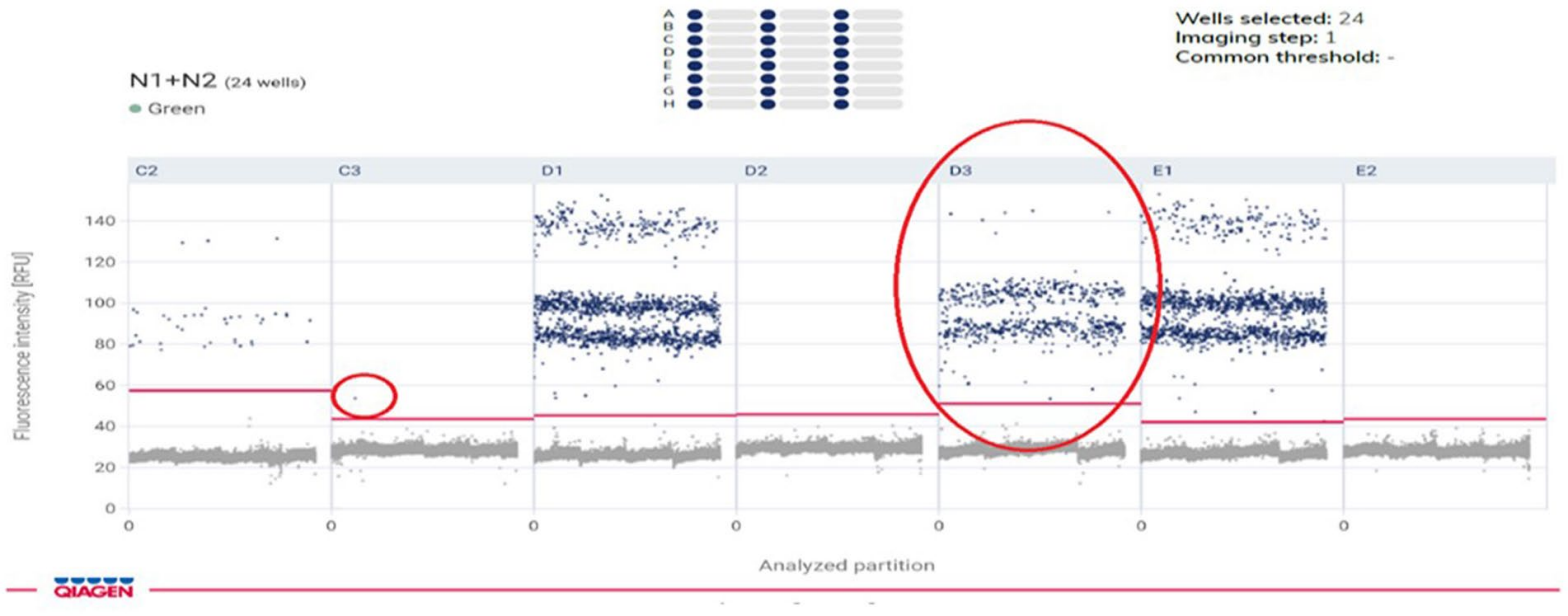
Amplitude signal of SARS-CoV-2 Ngene positive droplets obtained with DD-PCR.

A total of 214-paired samples from the 107 confirmed patients were tested by both qRT-PCR and ddPCR, including stool and urine sample. According to the qRT-PCR results, 106 samples were positive for stool and one for urine by N-gene. The ddPCR results of the 106 positive stool samples were also positive, and the Ct value of qRT-PCR was highly correlated with the copy number determined by ddPCR (N-gene, R2 = 0.89; N, R 2 = 0.20). In 107 patients, all the stool samples showed 99.06% positive concordance by both methods. Among the 106 negative urine samples identified by qRT-PCR, 77 (72.6%) samples were negative by ddPCR, and 29 samples were positive (Table 1). The median for ddPCR of the copy number for stool and urine samples was 11.30 and 0 respectively, whereas lowest copy number detected in ddPCR for both stool & urine sample was 0.048 copies/μL. Statistically difference was observed in urine specimens by using two tailed analysis [*p* < 0.0001] (Figure 4).

**Table 1:** Comparative analysis of DD PCR and q-PCR in both stool and urine specimens from SARS CoV-2 positive patients.

	Stool		Urine	
	qRT PCR	DD-PCR	qRT PCR	DD-PCR
Positive	106	107	1	29
Negative	1	0	106	78

Our findings demonstrated that whereas ddPCR performed better at detecting samples with low viral loads, like urine, qRT-PCR was equally as accurate and reliable in the identification of viruses from stool samples.

## 4. Discussion

From the beginning of the pandemic, qRT-PCR was used all over the world for the detection of the virus. During the most recent pandemic, qRT-PCR was regarded as the gold standard for virus detection. However, the failure of qRT-PCR in some cases in detecting the genes encoding the spike protein is a matter of concern. Besides that, qRT-PCR is also unable to quantify the viral load from borderline samples. Recent study showed that detection of COVID-19 virus form wastewater treatment by targeting the N1 and N2 coding genes showed positive results (11). Studies of viral load detection from plasma (12), nasopharyngeal swab (13), and sputum (14) showed that ddPCR is more sensitive in detecting the virus in comparision to qRT-PCR. However, this type of comparative studies until have not been reported from stool or urine samples.

The COVID-19 pandemic caused by the SARS CoV-2 virus motivates a variety of diagnostic strategies because of the novel causing pathogen, poorly known clinical consequence, and the limitation of testing resources. Furthermore, although the presence of SARS CoV-2 RNA in wastewater effluents has been established, viral infectivity of positive samples in cell cultures has not yet been established (15). SARS CoV-2 infectivity is sustained for more than 3 h in experimentally produced aerosols (16), and respiratory droplets and aerosols may contain high titers of virus particles (17–19). It should be noted that several research looking at viral shedding and faecal PCR in COVID-19 patients revealed a weak connection between positive stool PCR and level of gastrointestinal symptoms or disease activity (20). Furthermore, it is not yet known whether each stool PCR positive sample contains a live virus or only RNA pieces that have been discharged from the GI tract. Because of the variability in viral load across and within patients, it is crucial for diagnosis and surveillance to directly quantify absolute viral load from crude lysate. Here, in our study we look at the possibility of measuring SARS CoV-2 viral load using digital droplet PCR (ddPCR) directly from faecal and urine specimens. Using many partitioned reactions, digital droplet PCR quantifies the target nucleic acid sequences. Unlike qRT-PCR, which determines concentrations by comparing amplification rates to a standard curve, ddPCR cycles the sample to the endpoint and then counts target molecules directly by counting positive droplets. In comparison to qRT-PCR, this method offers a number of benefits, such as more accurate measurements and absolute quantification without the requirement for a standard curve (21, 22). The human immunodeficiency virus (HIV) (23), the cytomegalovirus (CMV) (24) and the human herpes virus 6 (HHV-6) (25) can all was detected using ddPCR. Purified RNA extracts used in ddPCR of COVID-19 patients show advantages for diagnosis and monitoring, especially in those with low viral loads (26–28).

One hundred and seven COVID-19 confirmed patients were tested to assess the viral load of SARS CoV-2 in stool and urine sample, and to measure the effectiveness of ddPCR in detecting the virus. For samples with high viral loads, we observed that both qRT-PCR and ddPCR provided reliable results; however, ddPCR performed better for those with low viral loads. It has been observed that the faeces contain higher viral load than the urine samples. Analyzing the stool and urine samples from 107 COVID-19 positive patients, we observed that ddPCR detects the virus with 100% concordance with qRT-PCR in the case of fecal specimens. While 29 urine samples out of 107 (27.1%) urine samples showed positive results in ddPCR, but qRT-PCR shows positive result for only two (1.86%) patients sample. These observations support that ddPCR is more sensitive in detecting the virus as compared to qRT-PCR. Although reverse transcription-PCR is sensitive and trustworthy, low-viral-load samples were more effectively detected by ddPCR in low viral load condition. Studies across the globe have identified the presence of live infective SARS CoV2 RNA particles in untreated sewage samples thus emphasizing the need for continuous environmental surveillance. Furthermore, it was observed that there is an association between the SARS CoV-2 RNA concentrations found in the water samples and the number of clinical cases reported in a particular area, thus implying that the surveillance of RNA concentrations of virus can be used as a potential early warning system to tract the community spread of the disease. There are several studies on the wastewater-based epidemiology (WBE) reported across the globe after the COVID-19 pandemic. WBE is being used globally to track SARS CoV-2 infections at the community level to aid public health responses to COVID-19. Regarding the sensitivity of WBE and its application in low prevalence situations, concerns still exist. Therefore, such assays will be good for monitoring infectious diseases, such as COVID-19, in the communities in the early stage. However, doing routine surveillance will not be an easy and cost effective task, since a huge number of pathogens are to be monitored regularly. The development of novel techniques in meta-genomics can be used in this regard for the simultaneous environmental surveillance of multiple pathogens, thus reducing the cost of such surveillance in resource poor settings.

The study was constrained by the small sample size for various types of samples and the fact that some patients did not have access to specific clinical information, which prevented results from being connected with symptoms or illness history. It is necessary to conduct further research on individuals who have comprehensive temporal and symptoms data as well as specimens that were collected sequentially from several sites.

## 5. Conclusion

Due to the diarrhoea symptom, stool is a more accurate signal of viral replication in the body along with throat and nasal swabs, and the viral load in stool samples tends to rise and then fall during the course of the illness. The COVID-19 pandemic spurred caused by the SARS CoV-2 virus sparks a variety of diagnostic strategies because of the novel causing pathogen, poorly known clinical consequence, and the limitation of testing resources. Because of the variability in viral load across and within patients, it is crucial for diagnosis and surveillance to directly quantify absolute viral load from crude lysate. Here, in our study we look at the possibility of measuring SARS CoV-2 viral load using digital droplet PCR (ddPCR) directly from fecal and urine specimens. Our study fills a gap of detection or the presence of SARS CoV-2 viral particles in urine samples that is a much easier specimen to get from patients than stools. We demonstrate that SARS CoV-2 standards can be properly quantified by ddPCR using pure RNA and a variety of sample matrices, including the widely used viral transport medium (VTM).

## Data availability statement

The raw data supporting the conclusions of this article will be made available by the authors, without undue reservation.

## Ethics statement

The studies involving human participants were reviewed and approved by ICMR-National Institute of Virology, Institutional Ethics Committee. The patients/participants provided their written informed consent to participate in this study.

## Author contributions

ML and MS contributed in the conceptualization and experimentation of the study. All the experimentation was done by MS, JR, NC, and PS. ML and JR did the data curation and analysis. MS and JR contributed in recruiting the patients. ML did manuscript writing, reviewing, and editing. ML also did supervision and project administration. All authors contributed to the article and approved the submitted version.

## Funding

This research was funded by ICMR-National Institute of Virology, Pune, Maharashtra, India.

## Conflict of interest

The authors declare that the research was conducted in the absence of any commercial or financial relationships that could be construed as a potential conflict of interest.

## Publisher’s note

All claims expressed in this article are solely those of the authors and do not necessarily represent those of their affiliated organizations, or those of the publisher, the editors and the reviewers. Any product that may be evaluated in this article, or claim that may be made by its manufacturer, is not guaranteed or endorsed by the publisher.
